# The effect of structured psychoeducation on caregiver burden in carers of patients with schizophrenia in Nigeria: A 12-week follow-up investigation

**DOI:** 10.4102/sajpsychiatry.v28i0.1703

**Published:** 2022-02-24

**Authors:** Theclar O. Iyidobi, Justus U. Onu, Obiora Iteke, Ngozi N. Unaogu, Richard Uwakwe

**Affiliations:** 1 Department of Psychological Medicine, Faculty of Clinical Medicine, University of Nigeria, Enugu, Nigeria; 2 Department of Mental Health, Faculty of Medicine, Nnamdi Azikiwe University, Nnewi, Nigeria; 3 Department of Clinical Services, Federal Neuropsychiatric Hospital, Enugu, Nigeria; 4 Department of Training and Research, Federal Neuropsychiatric Hospital, Enugu, Nigeria; 5 Department of Mental Health, Faculty of Medicine, Federal Neuropsychiatric Hospital, Enugu, Nigeria

**Keywords:** structured, psychoeducation, caregiver, burden, care as usual, schizophrenia, Nigeria

## Abstract

**Background:**

Despite robust evidence of the huge burden of caregiving amongst caregivers of patients with schizophrenia, there is a paucity of data in Africa on the interventions to address this enormous burden of caregiving.

**Aim:**

This study aimed to determine the effect of structured psychoeducation intervention on the burden of caregiving in comparison with ‘care as usual’ in a Nigerian Psychiatric Hospital.

**Setting:**

This study was done at the out-patient and in-patient units of the Federal Neuropsychiatric Hospital, Enugu, Nigeria.

**Methods:**

Caregivers of inpatients who fulfilled the International Classification of Diseases (ICD-10) criteria for diagnosis of schizophrenia were recruited for the study. The caregivers were then allocated into two groups (Group A received structured psychoeducation intervention in addition to ‘care as usual’ whilst group B received only ‘care as usual’). After the baseline assessment, the caregivers were followed up every 4 weeks for a period of 12 weeks. At each interval of follow-up, caregivers were assessed for caregivers’ burden using the Zarit Burden Interview (ZBI). Repeated measures analysis of variance (mixed type) was used to determine the effects of the interventions on caregivers’ burden in the two arms of the study across the intervals of follow-up.

**Results:**

The attrition rate at week 12 was 10.7%; leaving 130 for the assessment of outcome variable at the end of follow-up. Structured psychoeducation intervention was significantly better than ‘care as usual’ in ameliorating caregivers’ burden [F (1, 123) = 21.75, *p* < 0.001, Partial Eta Squared = 0.39].

**Conclusion:**

These findings seem to suggest that caregivers who received structured psychoeducation intervention experienced a greater reduction in caregiver burden than those who received ‘care as usual’. Whilst the study addressed short-term effect, the findings of this study are in accord with other studies that have supported the impression that psychoeducational family-based intervention is useful with regard to caregiver burden.

## Introduction

Schizophrenia is a disorder with varied pathophysiology and heterogeneous treatment outcome across cultures.^1^ Its course is highly variable, but it is often chronic with frequent relapses in the patients leading to increasing disabilities. Thus, the disorder has a significantly high cost to the patient in terms of personal suffering, on caregivers as a result of the shift of the burden of care from hospital to families and on the society in terms of direct, indirect and hidden cost from hospitalisation, lost productivity and stigma.^2^ This is even more important in low- and middle-income countries (LMICs) where there are no national social welfare programmes or insurance schemes for patients with severe mental disorders; thus families and relatives of most patients with schizophrenia bear the major burden of mental illness.^2^ Dillehey and colleagues defined the term ‘burden of care’ as a psychological state that ensues from the combination of physical, emotional, work and social pressure, such as economic restrictions that arise from taking care of a patient.^3^ A large body of evidence from both developing and developed countries agree that the burden on caregivers of patients with psychiatric disorders is enormous.^2,4,5^ In Nigeria, Lasebikan et al.,^6^ Adeosun,^7^ Inogho et al.,^8^ Okafor et al.^9^ and Yusuf et al.^10^ have reported on the burden and correlates of caregiver burden amongst carers of patients with schizophrenia. Lasebikan et al.^6^ and Okafor et al.,^9^ for example, found that the prevalence of objective and subjective caregiver burden in Nigeria ranges from 85.3% to 90% and 84.2% to 88%, respectively. In addition, the common correlates reported were severity of patients symptoms, number of hospitalisations, number of relapses, monthly income of caregivers, caregivers level of education and employment status, patients’ functional status, duration of illness and being a female caregiver.^6,7,8,9,10^ These authors agreed that there is need for a comprehensive intervention to reduce the burden of caregiving amongst carers of patients with chronic illnesses such as schizophrenia.^6,7,8,9,10^ Some authors have proposed that one remedy for the situation would be to provide support to the families by implementing formal family interventions that are culturally congruent, socially appropriate, economical and widely applicable.^2,5^ There seems to be widespread support for this recommendation with the need to support families by way of standardised family intervention packages (e.g. structured psychoeducation) being voiced by some Nigerian authors.^2,5^

Improving the burden of caregiving for caregivers of patients with schizophrenia has been a challenge to mental health service providers.^11^ In the past decades, many psychosocial interventions for supporting caregivers of patients with a mental disorder have been developed. One such programme is psychoeducation, which has been defined as a strategy of teaching patients and families about disorders, treatments, coping techniques and resources.^12^ Other psychosocial interventions with evidence of effectiveness in schizophrenia include social skills training, cognitive behavioural therapy, cognitive remediation and social cognition training amongst others.^13^ Psychoeducation may be looked at as the application of psychological theories and practices to educate people to help them have more information to tackle their challenges more effectively.^14^ By teaching skills such as problem solving and communication, caregivers’ coping abilities would increase.^15^

A large body of controlled evidence has unequivocally demonstrated the efficacy of structured family-based intervention for schizophrenia patients and their caregivers.^11,12,16^ These studies demonstrate that a relatively simple psychoeducation approach can be implemented in routine clinical settings and can have important effects. Structured psychoeducation intervention has emerged as superior to ‘care as usual’ in almost all studies.^17^ Thus, significant positive changes following structured family intervention have been observed in several areas including relapse and re-hospitalisation rates, psychotic symptoms, medication adherence, social functioning, relatives’ knowledge and attitudes, family burden, caregiver’s perception of support and their sense of self-efficacy.^18^ Progress made in the field of formal family interventions in the Western countries has not been replicated in developing countries such as Nigeria. This is important because of the differences in these settings. Firstly, mental health services in Western countries is accessed as close to the community as possible, whereas patients and carers in Nigeria travel long distances to access services in already congested tertiary centres.^19,20^ Secondly, payment for mental health services in Nigeria is by out-of-pocket expenses by patients and their family caregivers unlike the wide insurance coverage in the developed countries.^21^ Thirdly, with the decline in support from the extended family, this support was thought to cushion the effect of caregiving in the traditional African society.^22^ The primary aim of this study was to compare the effectiveness of a structured psychoeducational intervention for caregivers of patients with schizophrenia, with ‘care as usual’ on caregivers’ burden.

## Materials and methods

### Study design and population

This was a longitudinal follow-up intervention study. The study was carried out amongst caregivers of patients with a diagnosis of schizophrenia at the Federal Neuropsychiatric Hospital (FNH), Enugu, Nigeria. Treatment of schizophrenia in the centre is usually multidisciplinary (i.e. involving psychiatrists, psychiatric nurses, clinical psychologist, social workers and occupational therapist) and multimodal (i.e. involving the use of pharmacotherapy, psychotherapy and occupational rehabilitation). ‘Care as usual’ in this facility for caregivers usually involves unstructured psychoeducation given during ward rounds. According to statistics from the nurses’ record from January 2015 to December 2017, about five new admissions with a diagnosis of schizophrenia were made daily. The average length of hospitalisation was 10 weeks.

Participants were primary caregivers aged 18–60 years living continuously and were involved in the daily care of the patient for a year or more before the study. A primary caregiver was defined as someone who had been staying with the patient for some time, spending most time with the patient and intimately involved with his or her care.^23^ This entails looking after patient’s daily needs, supervising treatment, accompanying the patient to the hospital, and liaising with the treatment team. Caregivers currently providing care to another family member with (1) chronic physical illness or mental problems and/or (2) have personal history of any mental or chronic medical conditions at recruitment or (3) had received structured psychoeducation intervention from other agencies prior to the study were excluded.

### Sample selection

In computing the required sample size for the study, we used the formula by Wade [*n* = 2F(σ/d)^2^].^24^ This is because it uses constants in determining the minimum sample size and is culturally insensitive. Based on the power of 80% and 0.05% level of significance, the required sample size calculated was 70 per arm of the study after adjusting for attrition.

The list of the patient and caregiver pair already on admission before the commencement of the study was obtained from the nurses in the various wards. Thereafter, caregivers were balloted by picking from a basket with pieces of folded papers labelled ‘I’ and ‘C’. This was concealed in an envelope such that the nurse involved in the allocation and the caregivers do not know which group they belonged to. However, the researcher was aware which group the participants belonged to. Caregivers who picked a paper labelled ‘I’ were allocated to the intervention group (i.e. Group A), whilst those who picked the paper labelled ‘C’ were allocated to the care as usual’ group (Group B). However, the patient/caregiver pair who came into the ward after the study has commenced were made to pick from the basket as they presented. The intervention group comprised caregivers who received structured psychoeducation intervention in addition to the ‘care as usual’. The comparative group comprised caregivers who received ‘care as usual’ alone. ‘Care as usual’ was provided by the managing team and consisted of unstructured psychoeducation of the caregivers. Only caregivers caring for inpatients were recruited for the study as the period of admission allowed time for the required number of psychoeducation sessions. Contamination was partly prevented by allocation concealment. The details of sample selection is shown in Appendix 1.

## Procedure and measurement

### Diagnostic interview

The diagnostic interview of the patients was conducted by the researcher using the Mini International Neuropsychiatric Interview (MINI). The diagnosis was based on ICD-10 criteria for schizophrenia. Once the diagnosis of schizophrenia was confirmed, a thorough medical history and physical examination (including neurological examination) was carried out on the caregivers to exclude the presence of physical and psychiatric conditions. Caregivers were also screened for the presence of mental disorders using the MINI-Screening Version (MINI-screen). Thereafter, caregivers’ who met the inclusion criteria were given the socio-demographic and other study questionnaires.

### Questionnaires administration

The baseline assessment was performed in a convenient room in the ward. The caregivers of the experimental and the control group were given the self-administered Zarit Burden of Care Questionnaire to assess the baseline burden of caregiving. In addition, the baseline clinical status of the patients was also assessed using the Brief Psychiatry Rating Scale (BPRS) and Scale for the Assessment of Negative Symptoms (SANS), whilst the baseline functional status was assessed using the Social and Occupational Functioning Assessment Scale (SOFAS). This was repeated 4-weekly across the intervals of follow-up. A total of four assessments were carried out in the 12 weeks.

### Zarit Burden of Care Questionnaire

The Zarit Burden of Care Questionnaire was developed to assess caregiver burden in relatives of patients with chronic mental illness.^25^ It is a 22-item instrument that includes the factors most frequently mentioned by caregivers as problem areas in providing care for patients with mental disorders. It has a possible score of 0–88 depending on the caregiver responses. Responses are rated from 0 to 4, based on the level of distress. It has good internal consistency (Cronbach’s alpha value of 0.93).^26^ The instrument has been validated for use amongst informal carers in Nigeria.^27^

### Intervention

The caregivers of the experimental group received a structured psychoeducation intervention using a modified version of Sharif et al.^28^ (see Appendix 2).^28^ The majority of the sessions were carried out with 5–10 caregivers. This was performed at the occupational therapy hall of the hospital by the researcher and two of her assistants. The primary caregiver was required to attend all sessions. The structured psychoeducation intervention had 6 sessions delivered in 2 weeks. Three sessions in the first week and the remaining three sessions in the second week, with each session lasting for about 45 min. The sessions are delivered via lectures, group activities, questions and feedback. At the beginning of the sessions, attempts were made to build a positive therapeutic alliance with the caregivers. Preliminary information (oral/printed) about schizophrenia was provided. This was performed in a ‘no-fault atmosphere,: without attaching blame to anyone especially the caregiver. Caregivers in this group were encouraged not to divulge information to others during the period of the study.

The summary of the content of the psychoeducation manual was: education about the aetiology, symptoms, treatment, prognosis, discussion on medication management and alternative treatment. In addition, discussions on setting a realistic goal, substance abuse, marriage and related issues, communication, ways of providing positive and negative feedback, problem-solving training, identification of early signs of relapse, and employment opportunities were also part of the manual. All caregivers in the experimental group received this structured education in addition to care as usual offered by the managing team. The control group received only the care as usual as described earlier. The experimental group received the psychoeducation manual at the end of the psychoeducation session.

### Data analysis

Analysis of the result was performed using the International Business Machine-Statistical Package for the Social Sciences (IBM-SPSS-PC), version 20. Thereafter, the normality of distribution of data was checked using the Shapiro–Wilk test. It was found that the Zarit Burden score was normally distributed. Repeated measures analysis of variance (mixed model) was used to determine significant changes in the outcome variables (caregiver burden) whilst controlling for potential confounders such as caregivers’ employment status, the severity of the symptoms and psychosocial functioning of the patient. All tests of significance were at the 5% level of significance and confidence interval (CI) estimation of 95%.

### Ethical considerations

Approval for this study was obtained from the Ethics and Research Committee of the Federal Neuropsychiatric Hospital, Enugu, Nigeria, with reference number FNHE/HCS&T/REA/VOL.1/176. International ethical norms and standards were strictly adhered to. Written informed consent was obtained from all the participants. Participation was voluntary. In the process of the research, if any previously undiagnosed clinical condition was found in any of the caregivers, they were counselled and referred appropriately. As a result of the beneficial effect of structured psychoeducation on caregivers in the intervention arm; plans are on the way to commence it on the ‘care as usual’ arm of the study.

## Results

The caregiver participants of both arms of the study were similar in all socio-demographic characteristics as shown in Table 1. Table 2 shows that the caregivers in both arms of the study were similar with regard to their score in the caregiver burden scale at baseline (*p* = 0.08). After the 4th, 8th and 12th week, the ZBI score of the intervention group decreased more than the ‘care as usual’ group with good to excellent effect size (Table 2).

**TABLE 1:** Socio-demographic characteristics of the caregiver participants.

Variables	Cases (*n* = 70)		Control (*n* = 70)		Test-stat	*df*	*p*
	*n*	%	*n*	%			
**Age (SD) (years)**	44.58	12.43	42.90	15.13	*t* = -0.86	138	0.39
**Gender**					χ^2^ = 1.60	1	0.21
Male	19	28.8	13	19.4			
Female	47	71.2	54	80.6			
**Marital status**					χ^2^ = 4.66	2	0.10
Single	14	21.2	13	19.7			
Married	46	67.7	38	57.6			
Separated or divorced	6	9.1	15	22.7			
**Educational status**					χ^2^ = 2.89	3	0.41
No formal	3	4.5	6	9.2			
Primary	15	22.7	14	21.5			
Secondary	19	28.8	24	36.9			
Tertiary	29	43.9	21	32.3			
**Employment status**					χ^2^ = 0.07	1	0.79
Employed	58	92.1	56	93.3			
Unemployed	5	7.9	4	6.7			
**Religion**					0.28	-	-
Christianity	60	90.9	63	95.5			
Others	6	9.1	3	4.5			
**Relationship to patients**					0.12	1	0.73
Nuclear	49	77.8	53	80.3			
Extended	14	22.2	13	19.7			

Note: *N* = 140.

**TABLE 2:** Comparison of the effect of the intervention on caregiver and patient variables across intervals of follow-up.

Variable	Group	Mean difference	*t*-stat	*P*	Effect size
ZBIS T_0_	Intervention control	-2.18	-1.77	0.080	-
ZBIS T_1_	Intervention control	-3.73	-3.02	0.003	0.53
ZBIS T_2_	Intervention control	-6.54	-4.06	< 0.001	0.72
ZBIS T_3_	Intervention control	-9.03	-4.83	< 0.001	0.86

Figure 1 shows the effect of structured psychoeducation and ‘care as usual’ on caregivers’ burden between the two arms of the study. The mean ZBI score for the intervention versus the control were 52.70 versus 53.93 at baseline, 46.91 versus 50.80 at week 4, 44.20 versus 50.24 at week 8 and 41.52 versus 50.67 at week 12 [F(1, 123) = 21.75, *p* ≤ 0.001, Partial Eta Squared = 0.39]. Post hoc comparison shows that there was a significant difference in the reduction of caregiver burden between the intervention and the control group across all the intervals of follow-up. Figure 2 shows the effect of the intervention on caregivers’ burden whilst controlling for caregivers’ employment status, patients’ disease severity and functional status. There was a significant difference in the mean reduction of the caregiver’s burden score [F (1,112) = 12.71, *p* = 0.001, effect size = 0.10) between the two arms whilst adjusting for cofounders although with weak effect size.

**FIGURE 1:**
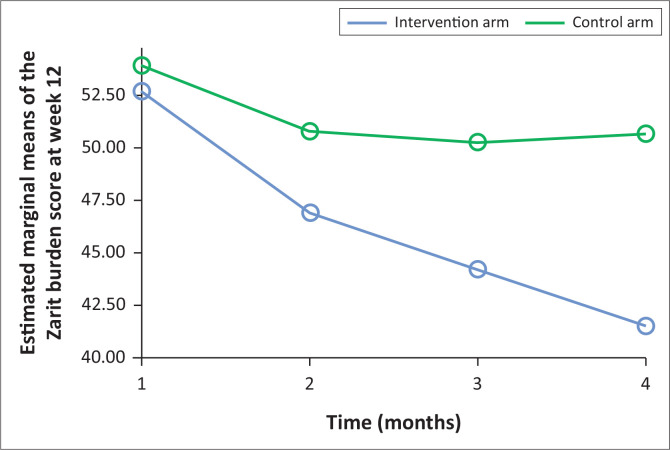
The effect of structured psychoeducation intervention and standard care on the caregiver participants’ burden of caregiving.

**FIGURE 2:**
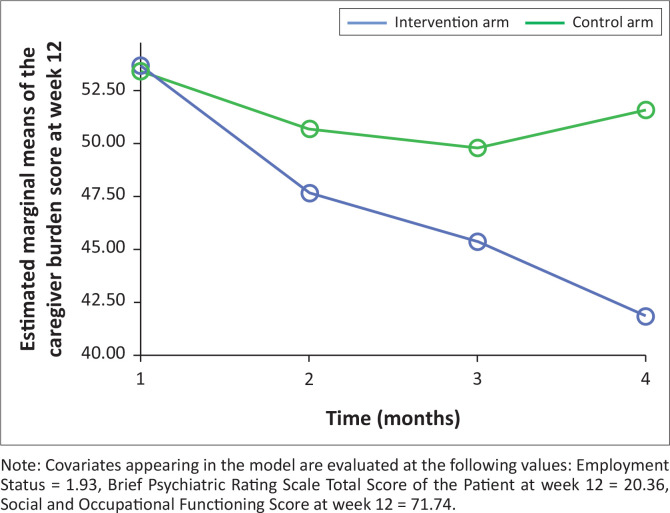
The effect of structured psychoeducation intervention and standard care on the caregiver participants’ burden of caregiving after controlling for potential cofounders.

## Discussion

The study aimed to determine the effectiveness of a structured psychoeducation intervention for caregivers of patients with schizophrenia, compared with ‘care as usual’ on the burden of caregiving.

Informal caregiving is an integral part of the care of people with severe mental illness, but the support needs of those providing such care are not often met.^29^ Carers focused intervention appear to improve the experience of caring and quality of life and reduce psychological distress of those caring for people with severe mental illness.^29^ The major finding of this intervention study is that caregivers who received structured psychoeducation intervention had significantly decreased the burden of caregiving when compared with those who received ‘care as usual’ at the end of follow-up. This effect remained even after controlling for potential confounders such as employment status of the caregiver, severity of symptoms and psychosocial functioning of the patient. This result is consistent with previous reports that psychoeducation improves caregivers’ burden.^29,30,31^ For example, Yesufu et al.^29^ reported improvements in the experience of caring and quality of life and reduced psychological distress of those caring for people with severe mental illness after psychoeducation.^29^ The mechanism by which psychoeducation intervention reduces caregivers’ burden is not clear. However, some authors have suggested that it does so through the indirect action of increasing knowledge, adherence and reduction of relapse and rehospitalisation.^17,32^ For example, Janet et al.^31^ reported that psychoeducation intervention reduced caregivers’ burden through improvement in coping skills and enhanced empowerment in dealing with crises amongst caregivers of patients with schizophrenia.^31^ Similarly, Mannion et al.^33^ reported improvement in coping strategies, personal distress and negative attitudes towards the affected relative in spouses of persons with serious mental illness after psychoeducation programme.

Contrary to the findings of the present study, some authors have reported no superior advantage between structured psychoeducation intervention and ‘care as usual’ in reducing caregivers’ burden.^16^ In a recent randomised-controlled trial on the impact of structured psychoeducation on patient and caregiver variables, Kulhara et al.^34^ found that structured psychoeducation intervention did not significantly reduce caregivers’ burden or improve caregivers’ coping skills. These authors argue that knowledge about the illness for a caregiver alone is not sufficient in reducing the caregiver burden.^17,35^ In other words, other confounding variables such as caregivers’ factors (e.g. level of education, employment status and time spent on the patient) and patients’ factors (i.e. the severity of symptoms and the functionality of the patient) are important mediators of caregivers’ burden.^17,32,36^ This is indeed the case with the present study. When other variables such as caregivers’ employment status, patients’ severity of illness and functionality were controlled, the effect of psychoeducation remained significant although with a weak effect size. Thus, comprehensive carers’ interventions should be multidimensional: addressing both patients’ and caregivers’ difficulties.

## Limitations

Firstly, the study was limited to only 12 weeks, a longer period of follow-up would have enquired into the sustainability of the interventions with time. Secondly, the use of carers within the community as against carers of inpatients would have given a real-world picture of the effects of psychoeducation on carers’ burden. Thirdly, although not significant, it appears that the intervention arm had lower scores in the caregiver burden scale at the baseline.

Despite the given limitations, the study has some strengths. Firstly, the longitudinal design of this study allowed for multiple examination of the changes in caregivers’ burden over time. Secondly, the attrition rate was low (10.7%) compared with 34.6% reported by Onu et al.^37^ in the same environment. Thirdly, the use of randomisation is still the gold-standard for such an intervention study and makes the result generalisable.

## Conclusion

This study has provided preliminary evidence of the superior efficacy of structured psychoeducational intervention compared with ‘care as usual’ for South Eastern caregivers of patients with schizophrenia.

## Figures and Tables

**FIGURE 1-A1:**
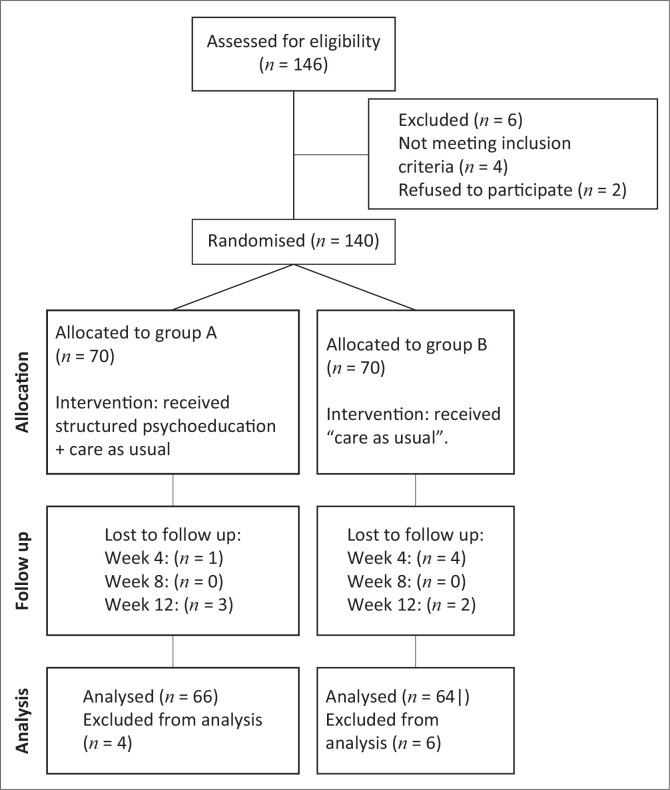
Consort Diagram.

## Appendix 2

**TABLE 1-A2:** The structured psychoeducation intervention using a modified version of Sharif et al.^28^

Session	Goals	Content
1	To orient caregivers to the programme and to create a trusting relationship between caregivers and instructors	Assessment of family needs.
		Overview of the programme and introduction of instructors and members to each other. Discussion of the importance of orientation to patient behaviours and symptoms. Presentation of a patient with a DSM-IV diagnosis of schizophrenia with clinically stable status. The patient describes his or her experiences and offers insight. The instructor offers explanations of the symptoms and behaviour, and of their effects on the family. Discussion of the aetiology and treatment. Discussion about warning signs of relapse. Explanation of the family role in relapse prevention. Exploration of the family intervention when the relapse has occurred. Question-answer session and group discussion.
2	To recognise the effect of medications and compliance	A review of the previous session. Discussion of positive and negative effects of antipsychotic drugs and problems related to side effects. Emphasis on the importance of drug compliance and maintenance Question-answer session and group discussion.
3	To improve communication skills in the family and to understand effective way to express emotion	A review of the previous session. Discussion of the importance of effective communication skills in the family and the role of environmental stress as a risk factor for schizophrenia relapse. Discussion of skills for effective communication between family members. Exploring intense emotions towards the patient. Discussion of expressing emotion and emotional environment in the family. Discussion of how to cope with the patient’s negative emotions. Question-answer session and group discussion.
4	To manage the patient’s symptom and skills in coping with them	A review of the previous session. Discussion of effective communication skills with patients when they have symptoms. Discussion of token economy and negative reinforcement for managing patients’ symptoms. Explanation of skills for coping with some of the patients’ symptoms.
5	To orient caregivers to stress management in the family	A review of the previous session. Introduction of importance of stress management in the family. Discussion of ways to reduce stress. Practicing relaxation methods during sessions. Question-answer session and group discussion.
6	To orient caregivers to relaxation methods	A review and summary of the contents of past session. Practicing relaxation methods during session. Conclusion.
